# Functional complexity of hair follicle stem cell niche and therapeutic targeting of niche dysfunction for hair regeneration

**DOI:** 10.1186/s12929-020-0624-8

**Published:** 2020-03-14

**Authors:** Chih-Lung Chen, Wen-Yen Huang, Eddy Hsi Chun Wang, Kang-Yu Tai, Sung-Jan Lin

**Affiliations:** 1 Department of Biomedical Engineering, College of Medicine and College of Engineering, National Taiwan University, Taipei, Taiwan; 2 Department of Dermatology, Columbia University, New York, NY USA; 3 Genome and Systems Biology Degree Program, National Taiwan University and Academia Sinica, Taipei, Taiwan; 4 Department of Dermatology, National Taiwan University Hospital and National Taiwan University College of Medicine, Taipei, Taiwan; 5 Research Center for Developmental Biology and Regenerative Medicine, National Taiwan University, Taipei, Taiwan; 6 Graduate Institute of Clinical Medicine, College of Medicine, National Taiwan University, Taipei, Taiwan

**Keywords:** Hair follicle stem cell, Niche, Function, Alopecia, Alopecia areata, Lichen planopilaris, Androgenetic alopecia, Therapy

## Abstract

Stem cell activity is subject to non-cell-autonomous regulation from the local microenvironment, or niche. In adaption to varying physiological conditions and the ever-changing external environment, the stem cell niche has evolved with multifunctionality that enables stem cells to detect these changes and to communicate with remote cells/tissues to tailor their activity for organismal needs. The cyclic growth of hair follicles is powered by hair follicle stem cells (HFSCs). Using HFSCs as a model, we categorize niche cells into 3 functional modules, including signaling, sensing and message-relaying. Signaling modules, such as dermal papilla cells, immune cells and adipocytes, regulate HFSC activity through short-range cell-cell contact or paracrine effects. Macrophages capacitate the HFSC niche to sense tissue injury and mechanical cues and adipocytes seem to modulate HFSC activity in response to systemic nutritional states. Sympathetic nerves implement the message-relaying function by transmitting external light signals through an ipRGC-SCN-sympathetic circuit to facilitate hair regeneration. Hair growth can be disrupted by niche pathology, e.g. dysfunction of dermal papilla cells in androgenetic alopecia and influx of auto-reacting T cells in alopecia areata and lichen planopilaris. Understanding the functions and pathological changes of the HFSC niche can provide new insight for the treatment of hair loss.

## Background

Hair forms a barrier to protect skin from external insults as well as to keep the body from temperature loss. Human hair, especially human scalp hair, also has important ornamental functions that are essential for social communication and senses of well-being. Unwanted hair loss can pose psychosocial distress to affected individuals [1]. Hair regeneration depends on the activation of hair follicle stem cells (HFSCs) [2–4]. As the hair follicle (HF) is an integral part of skin [5], its growth and the activity of HFSCs are regulated by various nearby cells of the HFSC niche in the skin [6, 7]. We categorize the component cells of the HFSC niche into 3 groups according to their functions, including signaling, sensing and message-relaying. We review how HFSC activity is regulated by different signaling cells and how sensing and message-relaying cells help HFs to initiate a regenerative attempt in face of local injury and external environmental changes. In diseased states, we discuss how the pathological changes of the niche lead to dysregulated hair growth. In addition, we discuss how the influx or emergence of non-preexisting cells within the HFSC niche affects hair growth and depletes HFSCs. We also highlight the therapeutic implications of niche pathology with an aim to prevent hair loss and to promote hair growth.

### Hair follicle structure, hair cycle and HFSC

**Fig. 1:**
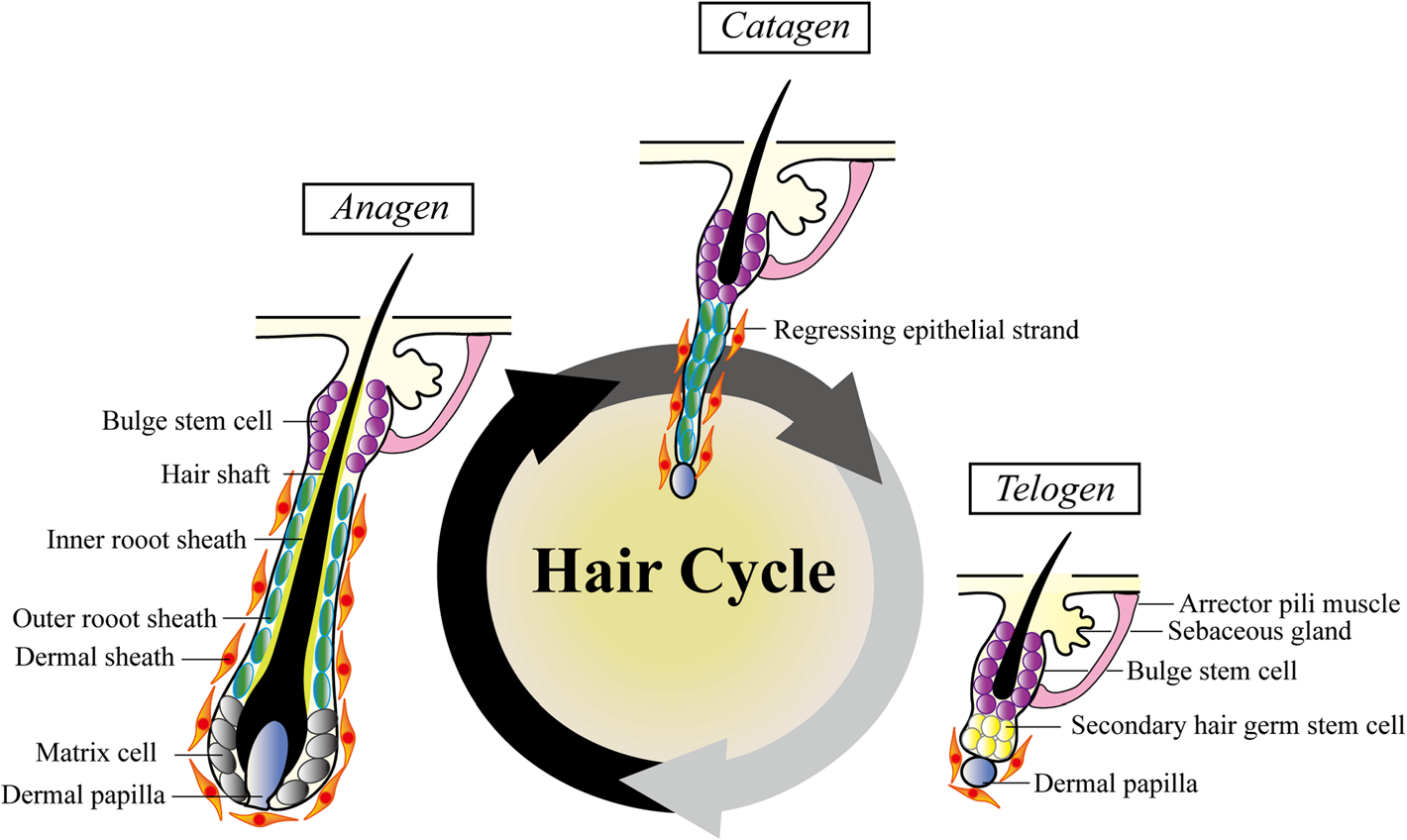
Hair follicle structure, hair follicle stem cell and hair cycle. Quiescent HFSCs reside in the bulge region and primed HFSCs are located in the secondary hair germ. They are transiently activated in early anagen, giving rise to progeny that grow down to form the lower portion of HFs. HFs progress through catagen (regressing phase), telogen (resting phase) and anagen (growing phase) cyclically. Matrix cells in the hair bulb actively proliferate and differentiate to support the continued elongation of the hair shaft in anagen. In catagen, the hair bulb shrinks and the lower portion of the HF regresses through a progressively shortened epithelial strand into the telogen HF. In telogen, HFSCs in the secondary hair germ and bulge remain inactivated

The HF is one of the few organs that undergo cyclic involution and regeneration throughout life [5, 6, 8, 9]. Structurally, HF is an epithelial organ consisting of two main parts: an epithelial cylinder composed of keratinocytes and the mesenchymal cells of dermal papilla (DP) and dermal sheath (Fig. 1) [5, 10]. During the hair cycle, HFs progress through anagen (growth), catagen (involution) and telogen (resting) phases and then re-enter anagen (Fig. 1) [5, 8–11]. Postnatal cycling and regeneration of HFs depend on sophisticated reciprocal epithelial-mesenchymal interaction [6, 12–19].

Over the past 3 decades, progress has been made in understanding how the growth of HFs is regulated, particularly due to the discovery of HFSCs [2–4, 20–22]. HFSCs are first identified as slow-cycling label-retaining cells located in the bulge epithelium [2, 22]. In addition to this population of relatively quiescent stem cells, HFs harbor another population of primed stem cells with faster activation dynamics in the secondary hair germ of telogen HFs [3, 5, 23]. HF regeneration from telogen to anagen is fueled by the coordinated activation of these two cell populations: primed HFSCs in the secondary hair germ are first activated, followed by the activation of quiescent HFSCs in the bulge later [2–5].

### Signals and signaling cells within HFSC niche

**Fig. 2:**
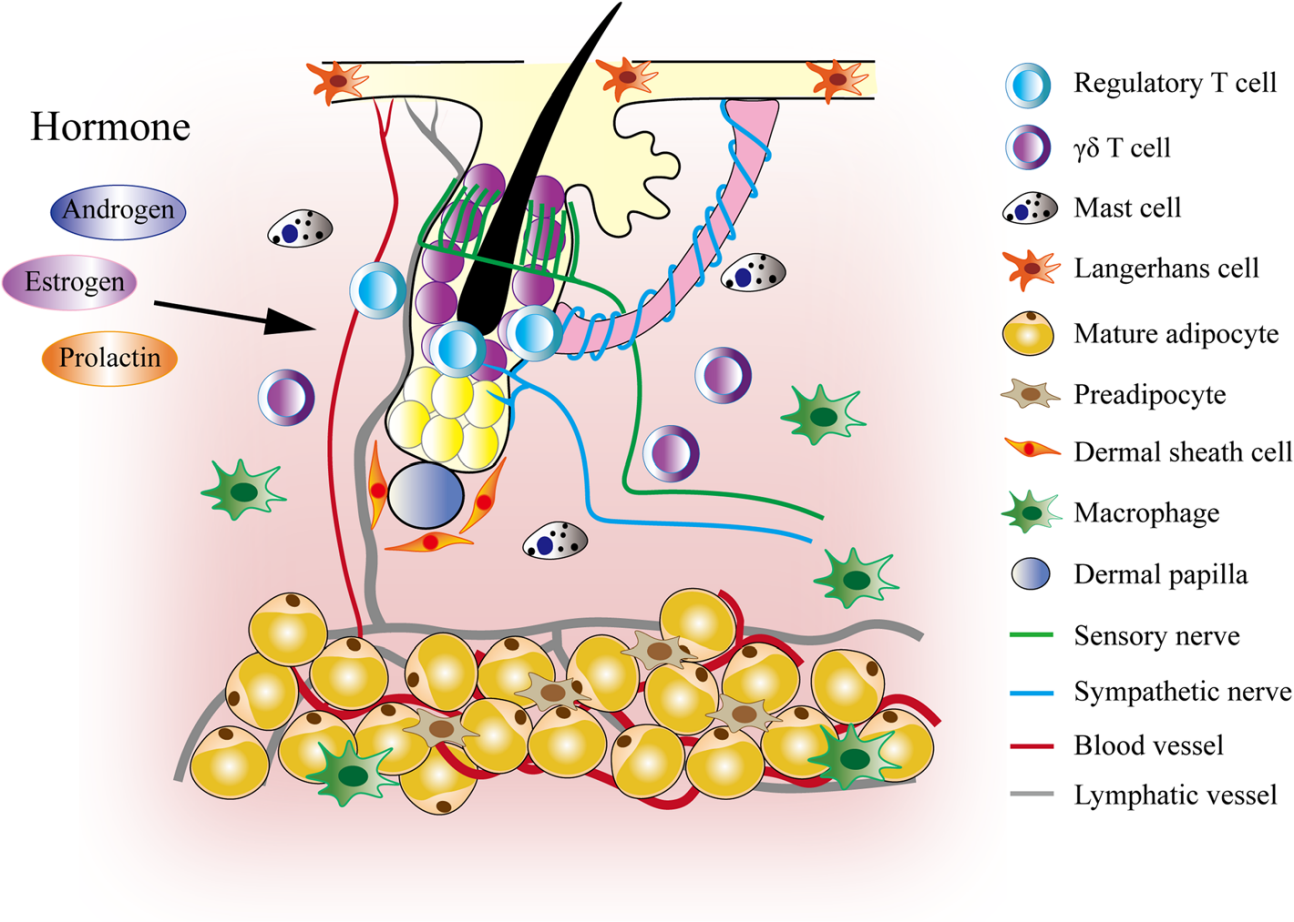
Hair follicle stem cell niche. The HFSC niche is composed of various component cells, such as dermal papilla, preadipocytes, adipocytes, immune cells and nerves. Systemic hormones also regulate HFSCs directly or indirectly through the HFSC niche cells. Both activating and suppressive signals are present within the HFSC niche. The probability of HFSC activation depends on the summation of all the activating and inhibitory signals

By definition, HF itself does not require the existence of surrounding niche cells to become a HF [24]. However, the integration of a variety of surrounding niche cells confer emergent functions on HFSCs, especially its ability to respond to changes of local, systemic and even external environments to begin a regenerative scheme or to remain quiescent. In diseased states, pathological infiltration of non-preexisting cells in the HFSC niche can lead to dysregulated hair growth. What constitutes the microenvironment that regulates HFSC activity and hair growth? Due to the continuous advance in hair research, more and more cell types (Fig. 2), including DP cells, adipose tissue, lymphatic vessels, nerves and immune cells, are identified to be contributing to the HFSC niche [15, 16, 25–36], unveiling the complexity and sophistication in the interaction of HFSCs with its environment. Since activating and inhibitory signals can both be present in the HFSC niche, the probability of HFSC activation is the readout of the summation of both activating and inhibitory signals [37, 38]. The two major counteracting signals are the bone morphogenetic protein (BMP) and Wnt/β-catenin signaling pathways [28, 37, 39]. High BMP signaling keeps HFSCs in an inactivated state, while Wnt/β-catenin signaling promotes HFSC activation and maintains HF growth [17, 28, 37–40]. Moreover, the TGF-β2, Foxp1 and oncostatin M signaling pathways have also been shown to regulate hair cycle [16, 29, 37, 41] . Factors that are able to tilt the balance of Wnt/β-catenin and BMP signaling can modulate HFSC activity, thereby suppressing or promoting anagen entry [6, 16, 29, 38, 42].

### Functional categorization- signaling, sensing and message-relaying modules in HFSC niche

**Fig. 3:**
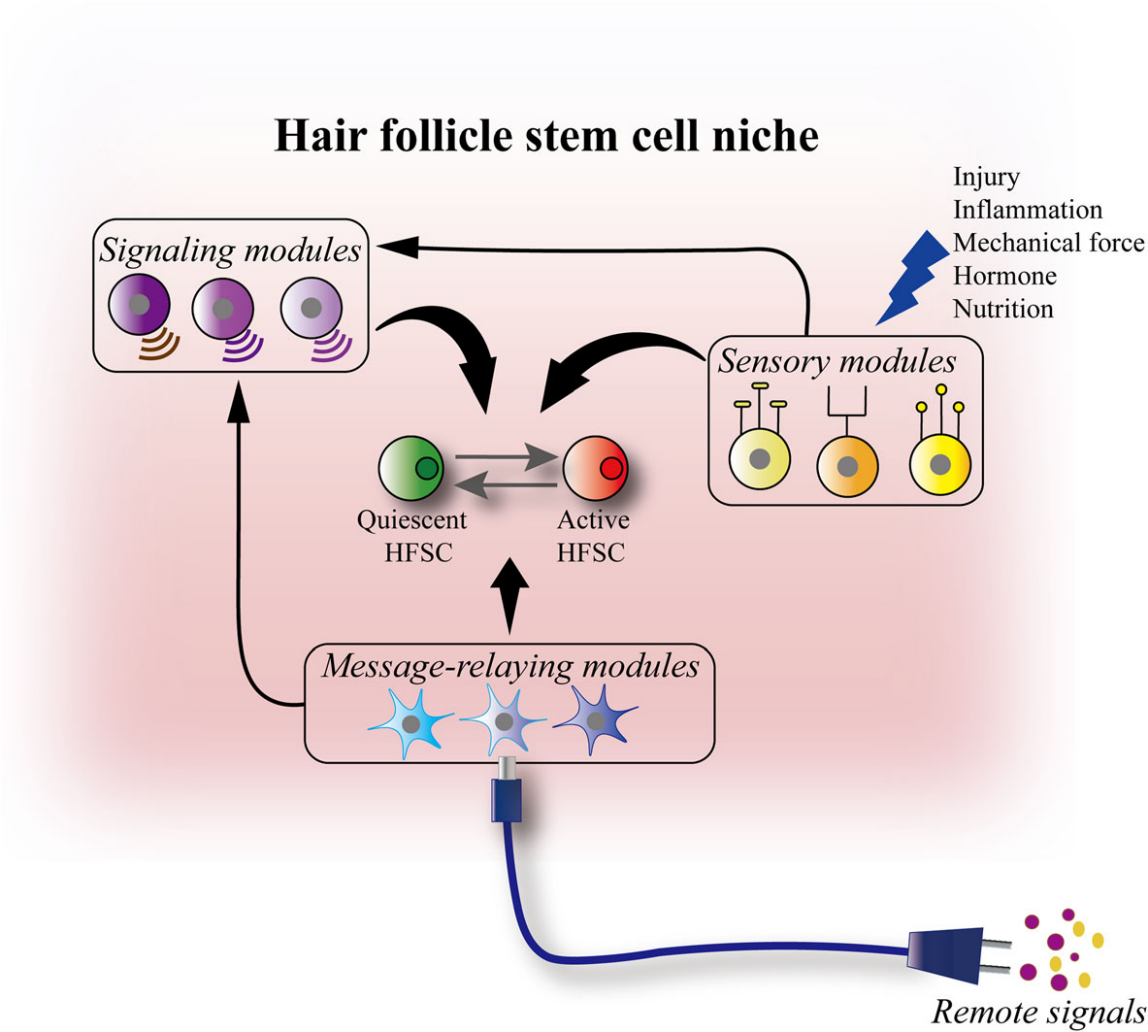
Functional categorization of HFSC niche cells. According to the functions of niche cells, they are categorized into 3 groups: signaling modules, sensing modules, message-relaying modules. These functionally distinct modules are assembled into a multifunctional niche. Signaling modules regulate HFSC activity via cell-cell contact or paracrine secretion. Sensory modules detect environmental cues. Message-relaying modules transmit signals from remote cells/tissues to HFSCs. Sensory modules and message-relaying modules can directly signal to HFSCs or indirectly regulate HFSC activity through the signaling modules

In addition to niche cells that provide either activating or inhibitory signals in the physiological state, niche cells of other functions also exist, enabling HFSCs to sense local, systemic and even external environmental changes to adjust their activity to meet local and organismal needs [28, 34, 35, 43–45]. Since additional cells of varied functions can be incorporated singly or in combination into the HFSC niche, we think that the niche cells can be modularized and these modules can be co-opted to construct the niche (Fig. 3). This is analogous to the design of a spaceship. The combination of different functional modules increases the functionality of the spaceship. Message-relaying modules allowed the communication between the Apollo 11 and NASA space center. The lunar module endowed Apollo 11 with an important function to land human on the moon. From this perspective, we divide HFSC niche cells into 3 functional modular categories: signaling, sensing and message-relaying (Fig. 3). Signaling cells directly provide activating or inhibitory signals for HFSCs through ligand secretion or cell-cell contact. Sensing cells detect the changes of local environmental cues and then directly or indirectly instruct HFSCs to remain quiescent or become activated. Message-relaying cells are capable of transmitting remote signals to the HFSC niche and then directly or indirectly modulate HFSC activity. Of note is that a single niche cell type can exhibit more than one function. We speculate that the co-option of various functional modules within a stem cell niche enables the animals to adapt their regenerative activity to the changing environment and to the altering physiological needs, thereby ameliorating the organismal fitness during evolution.

### Signals from dermal papilla for signaling and gain of testosterone-processing function in androgenetic alopecia

DP cells are an essential signaling component within the HFSC niche (Fig. 2). Epithelial-mesenchymal interaction is indispensable not only for embryonic HF morphogenesis but also for postnatal hair cycling [12, 13, 36, 46] . In the embryonic stage, interaction with the specialized mesenchymal niche of HF dermal condensate/papilla stimulates and instructs the epithelium to sequentially form placode, germ, and peg [46]. For postnatal follicular epithelial-mesenchymal interaction, although DP cells provide signaling ligands, such as TGF-β2 and FGF-7 [3, 16], to activate HFSCs for a new hair cycle, signals from epithelial cells are also required for proper anagen entry [17]. After HFs reach mature anagen, DP behaves as an instructive niche by regulating the proliferation and spatially ordered differentiation of transit-amplifying progenitors for proper hair shaft elongation and hair bulb structure maintenance [47]. Continued activation of Wnt/β-catenin signaling in DP through epithelial-mesenchymal interaction is indispensable for anagen progression [48]. During catagen, DP is also essential for the recession of the epithelial cylinder through controlled apoptosis/cell death [12]. In addition to physiological cycling, DP might also instruct the anagen repair process to avoid catagen entry when HFs are injured by chemo- and radiotherapy [49, 50].

Androgens are an important regulator for hair growth with paradoxical effects on HFs in different body regions. Androgens can stimulate the transformation of small vellus HFs into large terminal HFs after puberty, such as beard, pubic hair and axillary hair [51, 52]. On the contrary, in the scalp of genetically predisposed individuals of androgenetic alopecia or male pattern baldness, androgens inhibit hair growth, leading to progressive HF miniaturization [53]. Hyperandrogenism in females can lead to hirsutism with excessive male pattern hair growth [54]. These paradoxical effects of androgens on human hair growth have long been a puzzle [55, 56]. Androgens act through the intracellular androgen receptor. In HFs, androgen receptors are mainly expressed by DP [57, 58]. In contrast, keratinocytes do not express androgen receptors or show androgen receptor-dependent signaling activation, suggesting that keratinocytes may not be the primary responding cells in HFs [59, 60].

Alopecia due to HF aging is characterized by progressive HF atrophy with hair shaft miniaturization, prolonged telogen, and even loss of the entire HFs, resulting in diminished hair amount [61, 62]. In male, androgenetic alopecia is the most common disease of premature HF aging. With genetic predisposition in affected individuals, DP cells in the balding area exhibit higher activity of type II 5-alpha-reductase, an enzyme that are normally highly expressed in the prostate [58]. This enzyme converts testosterone into dihydrotestosterone through 5α-reduction of testosterone [57]. Dihydrotestosterone is a more potent androgen with a higher affinity than testosterone [63]. Local sustained dihydrotestosterone stimulation to DP compromises its functions, leading to deteriorating hair growth, shortened anagen and prolonged telogen [55, 56]. Therefore, the inappropriate gain of function, i.e. 5α-reducing ability to process testosterone, of these niche signaling cells in a patterned distribution is the primary cause of androgenetic alopecia.

DP cells from the balding scalp of androgenetic alopecia patients exhibit signs of senescent characters, such as loss of replicative potential, changes in cell size and shape, decrease or loss of is characteristic markers/molecular signature [64, 65]. Although the mechanisms are not fully clarified yet, dihydrotestosterone seems to induce premature senescence in DP due to persistent androgen receptor activation. The balding DP cells not only lose the ability to promote HFSC proliferation but also produce inhibitory factors that suppress HFSCs and disrupt keratinocyte proliferation [66–69]. For example, Wnt signaling is critical for anagen entry and anagen progression [17, 40]. Dkk1, a negative regulator of Wnt signaling, is overexpressed by balding DP cells [68]. Increased secretion of TGF-β1 from DP in catagen promotes anagen-to-catagen transition [12, 70]. TGF-β1 production is upregulated in balding DP and can compromise keratinocyte proliferation [69]. Additionally, balding DP cells also produce higher inflammatory cytokines, such as IL-6 [66, 67, 71]. IL-6 not only inhibits anagen entry but also disrupts normal anagen progression [66, 67, 71]. As a key mesenchymal signaling component in HFSC niche, targeted restoration of the normal signaling functions of DP cells can be an important strategy for the treatment of alopecia.

Currently, the most effective treatment for androgenetic alopecia is to suppress local dihydrotestosterone production by inhibiting 5-α-reductases. Finasteride and dutasteride are 5α-reductase inhibitors with different specificity and potency [72, 73]. Finasteride mainly inhibits the type-II 5α-reductase, the main 5α-reductase subtype in HFs, while dutasteride suppresses both type-I and type-II 5α-reductases. Long-term treatment with finasteride or dutasteride promotes hair growth in patients with androgenetic alopecia [74, 75]. Another FDA-approved medication for treating baldness is minoxidil [76, 77]. Minoxidil is a potassium channel opener originally designed for the treatment of hypertension [78]. Though the mechanisms are still unclear, it is speculated to promote hair growth through its effects on blood vessels or potassium channels [79].

### Mast cells, regulatory T cells, dendritic epidermal T cells

The HF maintains its own distinctive immune system, and the interplay between HFs with immune cells ensures proper hair growth and protection against autoimmunity [30, 35, 80, 81]. The immune cells, including macrophages, mast cells, and T cells, modulate the activity of HFSCs (Fig. 2) [30–32, 35]. Mast cells are found in the perifollicular compartment of the HF [80]. While the role of mast cells in HFSC activation and differentiation is still unclear, histochemical and ultrastructural analysis in the murine skin showed a high level of degranulation during late telogen to early anagen transition and late anagen to early catagen transition [32, 82]. Several molecules secreted by mast cells could contribute to HF turnover, including histamine and serotonin which promote epidermal keratinocyte proliferation in situ [83]. Mast cell activity is also suspected to contribute to hair loss disorders, such as androgenic alopecia and cicatricial alopecia [84–86].

Regulatory T cells (Tregs) have been shown to reside in the HFSC epithelium and are in close contact with HFSCs [31]. Tregs can augment HFSC proliferation and differentiation following hair plucking injury through Jagged1 (Jag1, 31]. The roles of Langerhans cells and dendritic epidermal T cells (DETC; γδ T cells), which are skin-resident antigen presenting cells and T cells respectively, in modulating HFSC activities are less defined [80]. Langerhans cells and DETCs are found in the outer root sheath of HFs [80]. The roles of DETCs on HFSCs have been reported in the context of wound healing. Activated DETCs not only stimulate epidermal stem cell proliferation to accelerate wound healing [87], but also favorably promote HFSC activation for hair regrowth [88].

### Macrophages for signaling and for injury- and force- sensing

Physiologically, clusters of skin-resident macrophages can be found in the perifollicular compartment and have been implicated in the regulation of hair cycles [30, 89]. The number of skin-resident CD11b^+^F4/80^+^Gr1^−^ macrophages decreases due to apoptosis prior to the onset of anagen [30]. Upon their apoptosis, they release stimulatory factors, such as Wnt7b and Wnt10a, which promote HFSC activation and differentiation [30]. More recently, it was reported that a different subset of TREM2^+^ dermal macrophages (trichophages) have an inhibitory effect on hair growth [29]. This study stemmed from the discovery that inhibition of JAK-STAT signaling promoted hair growth via disrupting the maintenance of HFSC quiescence [90]. Mechanistically, oncostatin M acts upstream of JAK-STAT5 signaling to maintain HFSC quiescence and oncostatin M is produced by TREM2^+^ macrophages [29]. Depletion of this specific subset of macrophages leads to premature anagen entry [29].

Macrophages also exhibit other functions, including the sensing of skin injury and mechanical force. Wounding promotes premature anagen entry in skin [44, 45]. When skin is wounded, macrophages are recruited and activated through the apoptosis signal-regulating kinase 1 (ASK1) [45]. Injury to HFs by hair plucking is also a potent stimulation to HFSCs. Injured by hair plucking, HFs recruit macrophages via the release of CCL2 [35]. TNF-α released by activated macrophages activates HFSCs by inducing AKT-dependent β-catenin accumulation [91]. Therefore, macrophages here capacitate the HFSC niche to sense the injuries to HFs or injuries to the surrounding skin to mount a regenerative attempt for skin protection. Additionally, macrophages also mediate the sensing of mechanical cues. Stretching skin can polarizes macrophages toward a M2 phenotype [92]. Pro-regenerative M2 macrophages stimulate hair regeneration via paracrine secretion of IGF and HGF. This demonstrates a mechanical force-macrophage axis in the regulation of tissue regeneration. Since there are multiple populations of macrophages within skin, each with distinct roles in the modulation of HFSC activation and differentiation, targeting macrophages can be a future direction for the management of hair loss.

### Influx of auto-reacting T-cells into HFSC niche disrupts hair growth in alopecia areata and lichen planopilaris

Proper HF cycling is strongly dependent on the homeostasis in the maintenance of HFSCs as well as intact immune privilege [81]. Collapse of immune privilege as a result of environmental factors or genetic predisposition puts HFs in risk of immune/inflammatory attack [81, 93, 94]. An active immune response with the secretion of inflammatory cytokines such as interferon-γ and TNF-α can certainly disrupt proper maintenance of HFSCs, leading to alopecia [94, 95]. These cytokines are secreted in abundance by lymphocytes that are not usually present in the physiological state, including CD4 and CD8 T cells (αβ T cells) that surround or infiltrate the HF [94, 96].

One of the most common immunity-mediated alopecia is alopecia areata. Alopecia areata is an autoimmune form of hair loss that may be patchy on the scalp or progress into total body hair loss [97]. Alopecia areata is reversible, indicating that HFSCs are not lost during autoimmune/inflammatory attacks [97]. However, the exact etiopathogenesis of alopecia areata has not been completely elucidated. The development of alopecia areata is associated with the collapse of HF immune privilege which subsequently increases antigen presentation to surveying T cells that recognize HF epithelial and/or melanocyte-associated antigens as foreign, and mount autoimmune responses against HFs [93, 97–100]. There are ongoing investigations trying to identify the exact HF antigen and antigen-specific T cells involved in the onset of alopecia areata [99, 101]. The autoimmune attack does not kill HFSCs specifically, but, instead, the lower transient portion of anagen HFs [97]. Since HFSCs are preserved, removal of these pathogenic T cells from HFSC niche restores hair growth. Due to the wide variation of clinical presentation, such as numbers and extent of lesions, age of onset, duration of disease persistence, and unpredictable responses to treatment, there is still a lack of universal guidelines for the treatment of alopecia areata. Topical or intralesional steroids are favored in patients with limited diseases and topical minoxidil can be employed as an adjuvant therapy [102–104]. In patients with extensive hair loss, systemic steroids and other immunosuppressants, such as methotrexate, can be considered. Additionally, immunotherapy with repeated topical application of contact sensitizers, such as diphenylcyclopropenone (DPCP), has also been employed in patients with extensive hair loss [102, 105, 106].

Dysregulation of Tregs have also been suggested to be associated with the collapse of HF immune privilege in alopecia areata [31, 107, 108]. A defect or lack of Tregs could lead to unchecked autoimmune attack on HF cells [108]. Improvement of alopecia areata was also reported (hair regrowth and reduction of CD4 and CD8 T cells) by treating the patients with low-dose IL2 to promote recruitment of Tregs into the skin [109].

Recently, it was shown that a population of CD8^+^/NKG2D^+^ T cells is necessary and sufficient for the development of alopecia areata [107, 110, 111]. The IFN-γ response and several γ-chain (γc) cytokines are significantly upregulated in alopecia areata skin which can activate cytotoxic CD8^+^/NKG2D^+^ T cell infiltration. Using anti-INF-γ antibody can efficiently block CD8^+^/NKG2D^+^ T cell infiltration and prevent alopecia areata development in the mouse model [110]. Through this research, the authors identified a small molecule inhibitor that can effectively block JAK-STAT signaling important for CD8^+^/NKG2D^+^ T cell function and reverse alopecia areata in both the mouse model and human patients [110]. This research subsequently led to two successful clinical trials repurposing FDA-approved JAK inhibitors, ruxolitinib (JAK1/2 specific) and tofacitinib (pan-JAK), to treat moderate to severe alopecia areata [112, 113]. Because of this, more clinical trials have started with the aim to optimize treatment of alopecia areata with JAK-specific inhibitors and different routes of administration [114]. As described above, inhibiting JAK-STAT signaling may have a direct impact on hair cycle. While alopecia areata-affected mice were treated, it was observed that the mice grew fuller hair. When the treatment was applied to wild-type mice at telogen, the mice entered anagen faster and grew fuller and darker hair [90]. These observations implicate a dual role of JAK inhibitors in alopecia areata by inhibiting CD8^+^/NKG2D^+^ T cells and promoting HFSC proliferation or differentiation.

Hair loss in lichen planopilaris, also a chronic inflammatory disease of HFs, is irreversible with a final scarring change [94, 115]. Lichen planopilaris usually runs a slowly progressive course, presenting with single or multiple patches of perifollicular erythema, scaling, follicular hyperkeratosis and eventual loss of HFs [116]. In contrast to alopecia areata in which cytotoxic T cells target the hair bulb, lichen planopilaris is characterized by Th1-biased cytotoxic T cell infiltration around the bulge region where HFSCs reside. It is postulated that a selective collapse of immune privilege in the HFSC niche, possibly triggered by interferon-γ, contributes to the pathogenesis of lichen planopilaris [94]. Chronic niche inflammation might deplete HFSCs by directly inducing HFSC apoptosis or indirectly altering the niche environment to a state unfavorable for the maintenance of HFSCs [94, 117]. Depletion of HFSCs leads to loss of entire follicular structures. Therapeutically, there is also a lack of consensus for the treatment of this disease. Current treatment mainly relies on immunosuppressants, such as topical, intralesional or systemic steroids, hydroxychloroquine, cyclosporine and mycophenolate mofetil [116, 118]. Prevention of the collapse of immune privilege of the HFSC niche can be a future direction for the treatment and prevention of this disease [94].

### Signals from adipose tissue and nutritional sensing

Dermal white adipose tissue is a highly dynamic tissue in skin and the thickness oscillates during hair cycles [27, 119–121]. The dermal white adipose tissue becomes thickened from telogen to anagen and then decreases in thickness from anagen to catagen transition [120]. The increase of dermal white adipose tissue thickness during telogen to anagen transition is mainly contributed by proliferation and differentiation of preadipocytes and hypertrophy of maturate adipocytes [27, 122]. The maturation of preadipocytes with increased adipogenesis is dependent on epidermal Wnt/β-catenin and sonic hedgehog (SHH) signaling [123, 124]. Epidermal Wnt/β-catenin signaling is a signaling cascade initiator that is required for dermal adipocyte differentiation [123]. After anagen is initiated, the increased production of SHH by HF transit-amplifying cells promotes adipogenesis in preadipocytes via peroxisome proliferator-activated receptor γ [124]. How the dermal white adipose tissue thickness is reduced during anagen to catagen transition is still unclear. Since no apoptosis of mature adipocytes is detected [27], it is possible that adipocytes might undergo dedifferentiation through a lipolytic or autophagic process [121]. During anagen to catagen transition, HFs express higher TGF-β1, which suppresses proliferation and increases apoptosis in HFs [125]. HFs might induce adipocyte dedifferentiation through TGF-β1 signaling in catagen [126].

Adipose tissue has been shown to exhibit non-metabolic functions [127]. In bone marrow, hematopoiesis and hematopoietic stem cell activity are suppressed when more mature adipocytes are present [128, 129]. In skin, adipocytes and preadipocytes show opposite roles in the regulation of HFSC activity during the physiological hair cycling (Fig. 2) [127]. Immediately after HFSCs are activated to initiate anagen growth, mature adipocytes release BMP proteins to suppress the activity of HFSCs [28]. This might prevent overactivation of HFSCs by consolidating quiescence. On the other hand, during the transition from telogen to anagen, preadipocytes stimulate HFSCs through paracrine secretion of PDGF [27]. The reciprocal signaling and intimate interaction between HFs and adipose tissue highlight the interdependence between HFSCs and its niche cells to maintain appropriate tissue dynamics in skin.

In addition to passive fat storage, adipose tissue also exhibits other non-metabolic functions [127]. During bacterial invasion, cutaneous adipocytes undergo reactive adipogenesis to increase the production of anti-microbial peptides against bacteria [130]. We speculate that adipocytes in the HFSC niche might play a role in sensing environmental changes, such systemic nutritional states or local skin injury. Hair growth is affected by the systemic nutritional states [131]. In human, impaired hair growth is observed in individuals with protein/energy malnutrition [132]. HFs can be arrested in prolonged telogen during experimental calorie restriction [5, 133]. How HFSCs detect the systemic nutritional states is unclear. One possibility is that HFSCs can directly sense the systemic nutritional changes. mTOR signaling is a key pathway for metabolic response to the nutritional state [134]**,** and upregulated mTOR signaling is essential for HFSC activation in the early anagen and regeneration following ionizing radiation injury [135, 136]. HFSCs might tune its mTOR signaling according to the changes of systemic nutrition. The other possibility is that the nutritional states are detected by niche cells, such as adipocytes. In the intestine, calorie restriction reduces mTOR activity in the niche Paneth cells [137]. Subsequently, Paneth cells signal to intestinal SCs to increase intestinal SC numbers. Clinical observation suggests that obesity might negatively affect hair growth [138]. Adipocytes might regulate HFSCs through the release of adipokines according the systemic nutritional states [127].

### Signals from sensory nerves and message-relaying function of sympathetic nerves to activate HFSCs via an ipRGC-SCN-sympathetic circuit

HF is a highly innervated sensory organ. The non-encapsulated endings of sensory nerve surround HFs for the mechanosensory function [139, 140]. In HFs, sensory nerves innervate upper bulge to form the sensory piloneural niche (Fig. 2) [139, 140]. Through secreting SHH ligands, this sensory piloneural niche maintains higher hedgehog signaling activity in the HFSCs of the upper bulge region [26]. Although this sensory piloneural niche does not significantly affect hair regeneration, the ability of the upper bulge cells to repair epidermal injury is dependent on the sustained upregulation of hedgehog signaling [26].

**Fig. 4:**
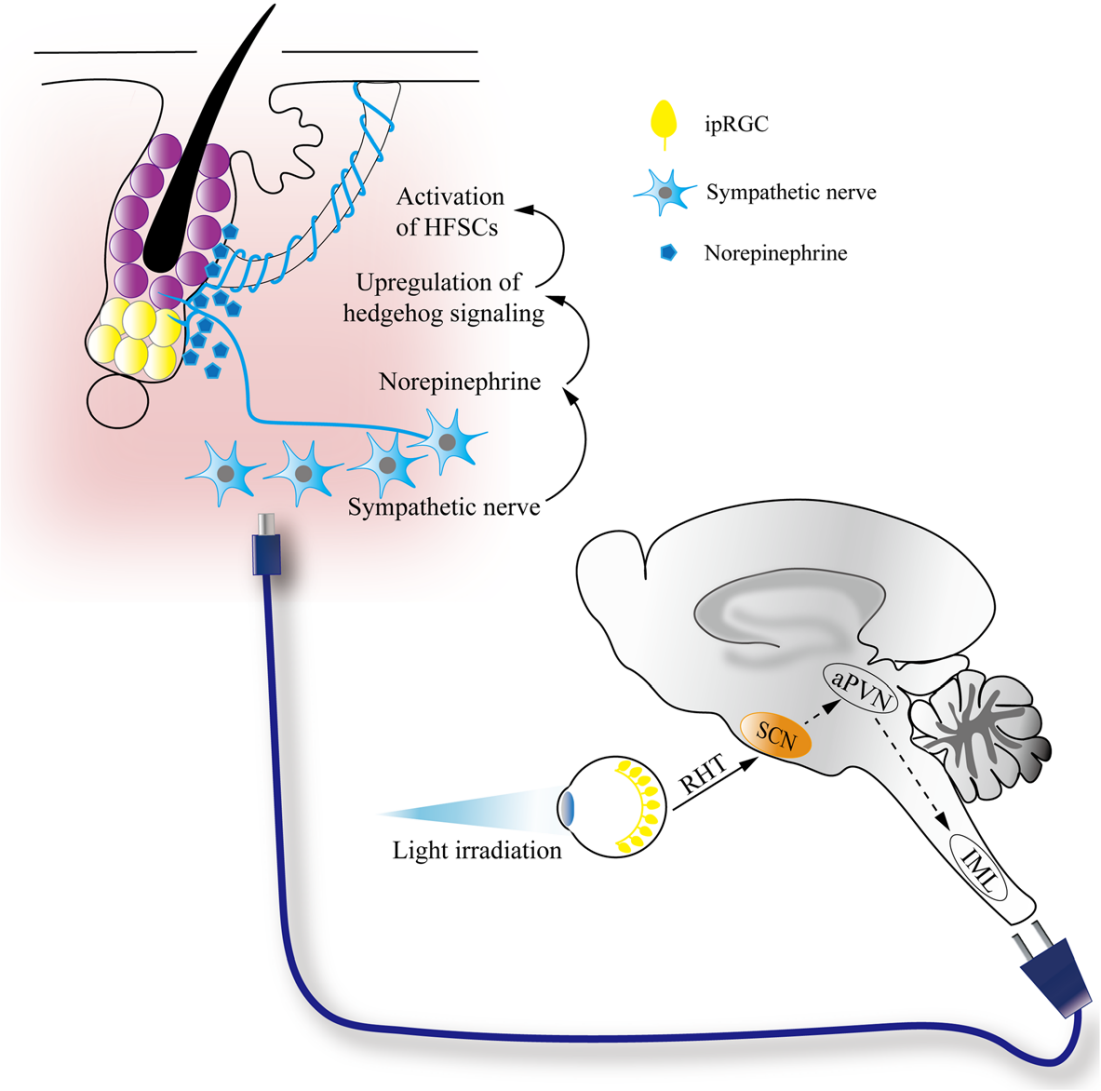
Sympathetic nerves relay external light signals to HFSCs. Sympathetic nerves are a gateway for the communication between internal HFSC niche and external environment. Intense light irradiation to eyes promotes HFSC activation through an ipRGC-SCN-sympathetic nervous circuit. Increased norepinephrine release from cutaneous sympathetic nerves facilitates HFSC activation by upregulating hedgehog signaling

The piloerection function of HFs relies on the ordered integration of sympathetic nerves and arrector pili muscle around HFs. Sympathetic nerves not only densely surround arrector pili muscle but also loop around HFSCs [34, 140]. It is intriguing whether sympathetic nerves around the HFs have dual roles in both piloerection (goosebumps) and HFSC regulation. In the bone marrow, sympathetic nerves control multiple functions of hematopoietic SCs, including their mobilization, maintenance of young functional signature and regeneration from chemotherapeutic injury [141–143]. Sympathetic nerves also transduce the central circadian rhythms to hematopoietic SCs for their daily rhythmic oscillating egress from the bone marrow [143–145]. Clinical observation showed that hypertrichosis in the form of “hemitrichosis” can be a result of sympathetic nerve hyperactivity due to thoracic surgical injury [145], suggesting a stimulating effect of sympathetic nerves to hair growth. Early experiments suggested that sympathetic nerves might promote anagen progression after HFSCs are activated in the physiological state [140]. We found that light can stimulate hair growth not only directly through cutaneous irradiation but also indirectly through the eyes [34, 43, 146]. Light irradiation to murine eyes, a danger signal to nocturnal animals, is detected by the non-conventional photoreceptor melanopsin of intrinsically photosensitive retinal ganglion cells (ipRGCs) (Fig. 4) [34]. Light signals are transmitted via ipRGCs to the suprachiasmatic nucleus to activate the systemic sympathetic system. A high sympathetic tone increases local norepinephrine release which subsequently upregulates hedgehog signaling in HFSCs, promoting their activation. Therefore, sympathetic nerves are the niche gateway for internal HFSCs to communicate with the external world by relaying the external light signals to the HFSC niche. Therapeutically, stimulating adrenergic receptors of HFSCs can be a way to promote hair growth.

## Conclusion

Since cyclic hair regeneration can be easily observed, the HF has become a favored model to explore how tissue stem cell activities are regulated. Accumulative results have helped to identify various component cells of the HFSC niche and to elucidate how these niche cells influence HFSCs. The integration of functional distinctive niche modules, such as signaling, sensing and message-relaying modules, has added the complexity of HFSC regulation and also allows HFSCs to interact with the local, systemic and external environments to adapt their activity for tissue needs. Pathological changes of the HFSC niche can lead to dysregulated hair growth or HFSC loss in diseased states. Studying how HFSCs are regulated by the niche in the physiological and diseased states can uncover new therapeutic targets to prevent hair loss as well as to promote hair regeneration.

## Data Availability

Not applicable.
